# Clinical profile and mortality in patients with *T*. *cruzi*/HIV co-infection from the multicenter data base of the “Network for healthcare and study of *Trypanosoma cruzi*/HIV co-infection and other immunosuppression conditions”

**DOI:** 10.1371/journal.pntd.0009809

**Published:** 2021-09-30

**Authors:** Maria Aparecida Shikanai-Yasuda, Mauro Felippe Felix Mediano, Christina Terra Gallafrio Novaes, Andréa Silvestre de Sousa, Ana Marli Christovam Sartori, Rodrigo Carvalho Santana, Dalmo Correia, Cleudson Nery de Castro, Marilia Maria dos Santos Severo, Alejandro Marcel Hasslocher-Moreno, Marisa Liliana Fernandez, Fernando Salvador, Maria Jesús Pinazo, Valdes Roberto Bolella, Pedro Carvalho Furtado, Marcelo Corti, Ana Yecê Neves Pinto, Alberto Fica, Israel Molina, Joaquim Gascon, Pedro Albajar Viñas, Juan Cortez-Escalante, Alberto Novaes Ramos, Eros Antonio de Almeida

**Affiliations:** 1 Departament of Infectious and Parasitic, Faculdade de Medicina, University of São Paulo, São Paulo, Brazil; 2 Laboratory of Immunology, Hospital das Clínicas, Faculdade de Medicina, University of São Paulo, São Paulo, Brazil; 3 WHO Technical Group IVb on prevention, control and management of non congenital infections of the Global Network for Chagas Disease Elimination, WHO, Geneva, Switzerland; 4 Evandro Chagas National Institute of Infectious Diseases, Oswaldo Cruz Foundation, Health Ministry, Rio de Janeiro, Brazil; 5 Division of Infectious Diseases, Hospital das Clínicas, Faculdade de Medicina, University of São Paulo, São Paulo, Brazil; 6 Division of Infectious Diseases, Department of Internal Medicine, Ribeirão Preto Medical School University of São Paulo, Ribeirão Preto, Brazil; 7 Discipline of Infectious and Parasitic Diseases, Department of Internal Medicine, Federal University of Triângulo Mineiro, Uberaba, Brazil; 8 Centre for Tropical Medicine, School of Medicine, University of Brasilia, Brasília, Brazil; 9 Federal University of Health Sciences of Porto Alegre, Porto Alegre, Brazil; 10 Hospital de Infecciosas, Buenos Aires, Argentina; 11 National Institute of Parasitology, Departament of Clinics, Pathology and Treatment, Health Ministry, Buenos Aires, Argentina; 12 Department of Infectious Diseases, Vall d’Hebron University Hospital, PROSICS, Universitat Autònoma de Barcelona, Barcelona, Spain; 13 ISGlobal, Universitat de Barcelona, Hospital Clínic, Barcelona, Spain; 14 Departamento de Medicina, Asignatura Enfermedades Infecciosas, Facultad de Medicina, Universidad Buenos Aires, Buenos Aires, Argentina; 15 Evandro Chagas Institute, Health Surveillance Secretary, Health Ministry, Belém, Brazil; 16 Facultad de Medicina, Universidad Austral de Chile, Valdivia, Chile; 17 Department of Control of Neglected Tropical Diseases, World Health Organization, WHO, Geneva, Switzerland; 18 Pan American Health Organization (PAHO), World Health Organization (WHO), Brasília, Brazil; 19 Department of Community Health, School of Medicine, Federal University of Ceará, Fortaleza, Brazil; 20 Department of Internal Medicine, Faculty of Medical Sciences, State University of Campinas, Campinas, Brazil; Tulane University, UNITED STATES

## Abstract

**Objective:**

Chagas disease (CD) globalization facilitated the co-infection with Human Immunodeficiency Virus (HIV) in endemic and non-endemic areas. Considering the underestimation of *Trypanosoma cruzi (T*. *cruzi)*-HIV co-infection and the risk of life-threatening Chagas Disease Reactivation (CDR), this study aimed to analyze the major co-infection clinical characteristics and its mortality rates.

**Methods:**

This is a cross-sectional retrospective multicenter study of patients with CD confirmed by two serological or one parasitological tests, and HIV infection confirmed by immunoblot. CDR was diagnosed by direct microscopy with detection of trypomastigote forms in the blood or other biological fluids and/or amastigote forms in inflammatory lesions.

**Results:**

Out of 241 patients with co-infection, 86.7% were from Brazil, 47.5% had <200 CD4^+^ T cells/μL and median viral load was 17,000 copies/μL. Sixty CDR cases were observed. Death was more frequent in patients with reactivation and was mainly caused by CDR. Other causes of death unrelated to CDR were the manifestation of opportunistic infections in those with Acquired Immunodeficiency Syndrome. The time between the co-infection diagnosis to death was shorter in patients with CDR. Lower CD4^+^ cells count at co-infection diagnosis was independently associated with reactivation. Similarly, lower CD4^+^ cells numbers at co-infection diagnosis and male sex were associated with higher lethality in CDR. Additionally, CD4^+^ cells were lower in meningoencephalitis than in myocarditis and milder forms.

**Conclusion:**

This study showed major features on *T*. *cruzi*-HIV co-infection and highlighted the prognostic role of CD4^+^ cells for reactivation and mortality. Since lethality was high in meningoencephalitis and all untreated patients died shortly after the diagnosis, early diagnosis, immediate antiparasitic treatment, patient follow-up and epidemiological surveillance are essentials in *T*. *cruzi*/HIV co-infection and CDR managements.

## Author summary

Chagas disease (CD) is a chronic infectious disease caused by the parasite *T*. *cruzi* that accounts for a major burden due to its life-lasting morbidity in endemic and non-endemic areas. Globalization facilitated the meeting of CD and Human Immunodeficiency Virus (HIV) infection (*T*. *cruzi*/HIV co-infection). Patients with this co-infection are at risk of life-threatening CDR. This study aimed to analyze the clinical characteristics and mortality rates of patients with *T*. *cruzi*/HIV co-infection.

This retrospective multicenter study included 241 patients with co-infection, of those 60 with CDR. Death was more frequent in patients with reactivation and was mainly caused by CDR. Our data showed that co-infection diagnosis is close to the reactivation diagnosis and characterized by poor immune response associated to CDR, higher lethality and more severe forms of CDR (meningoencephalitis). This study emphasizes the prognostic role of CD4^+^ cells on reactivation and mortality and the need of epidemiological surveillance for co-infection and CDR.

## Introduction

Chagas disease (CD) is a chronic infectious disease caused by *T*. *cruzi* that accounts for a major burden due to its life-long duration. It currently affects 6–7 million people not only in Latin America but also in different continents, mostly due to the population movements [1,2]. *T*. *cruzi* infected people is estimated in Europe in 68,000–120,000 [3], and in the United States (US) >300,000 [4]. The control of vectorial transmission (through triatomine bugs) has improved in most Latin American territories, but the challenge now persists with oral and congenital transmissions and with blood transfusions and organ transplantation wherever its full prevention coverage has not been reached [1,2].

The risk of CDR is increased in chronically immunosuppressed CD patients suffering from HIV infection/AIDS, neoplasia, autoimmune diseases, immunosuppression treatment and organ transplants [5,6].

The first case of *T*. *cruzi*-HIV co-infection was reported in 1990 [7], but CDR has been already cited in 1988, in a patient with AIDS [8]. Considering the worldwide estimated 38 million people living with HIV in 2019, a sustained risk of *T*. *cruzi*-HIV co-infection is still a reality in endemic and non-endemic areas for CD with infected immigrants [1,9]. In Brazil, the country with the highest estimated *T*. *cruzi* infected individuals (1.3–3.2 million in 2020), CDR was considered an AIDS defining condition since 2004 [10]. In WHO regions of America, an endemic region for CD, the same was defined in 2007 [11].

Co-infection cases have been reported predominantly in Brazil and Argentina, but also in Spain, USA, Chile, Colombia, Jamaica, Germany, Switzerland, Bolivia, Venezuela [5,7,12–23]. The prevalence of co-infection is estimated at 1.3%-2.3% in São Paulo State [5,24] and 5.0% in Rio Grande do Sul State [25] in Brazil and 4.2% in Argentina, varying from 1.3 to 10.5% in endemic areas [5,21,24–26] and in a restricted group of HIV infected immigrants from CD endemic areas [27], with higher rates in drug users in Argentina [21,26]. Estimates of *T*. *cruzi*-HIV co-infection cases range from 4,570–15,360, based on patients living with HIV in Brazil and Argentina [1,5,9,24–26] and in approximately 21,740 in Latin America [5].

Study on mortality from *T*. *cruzi*-HIV co-infection in Brazil using the mortality database from AIDS (103,000 deaths) and CD (about 54,000 deaths), with both diagnoses recorded in death certificates, showed that AIDS was the basic cause of death in 77.9% and CD in 17.6% [28]. Chagas Disease reactivation rates ranged from 10 to 15% among HIV co-infected people in retrospective case series [5,21] and was estimated to be about 10% in patients followed in a prospective study [6,15], needing to be better established in larger studies.

The most affected sites in CDR are the CNS (74.2%) and the myocardium (16.7%). Other affected sites in 9.2% of cases are the duodenum, skin, cervix, peritoneum, pericardium, stomach and eye tissue. Mortality rate was 73.4% in previous cases of reactivations [5]. In these cases, CD4^+^ levels were generally lower than 200/μL [5,10,15,21] with an average of 98/μL in reactivation and 562/μL without reactivation [5]. This clinical profile of CDR does not differ from reactivation secondary to other forms of immunosuppression, especially chemotherapy for malignant neoplasms and those related to organ transplants. The affected sites of CDR from these other forms of immunosuppression are the central nervous system and the heart, as occurs in *T*. *cruzi*/HIV co-infection in AIDS situations, with the same clinical outcomes [6,29]. Oligosymptomatic cases and/or panniculitis/erythema nodosum could occur frequently in solid organ transplants. It is important to note that other infections are more frequent in AIDS than *T*. *cruzi*/HIV co-infection, requiring a differential diagnosis [5]. Brainstem involvement on toxoplasmosis is more frequent than in *T*. *cruzi*/HIV co-infection, although CDR lesions can also occur in the brainstem, delaying the diagnosis and the specific treatment for *T*. *cruzi* [30].

An imbalance in the host-parasite relationship towards a Th2 response in case of co-infection, particularly in patients with patent parasitemia [31,32], has been postulated as one of the factors associated with bidirectional influences on the interaction between HIV and *T*. *cruzi* in placenta [33] and astrocytes cells [34], leading to increased parasite multiplication. Elevated parasitemia, presented in reactivation, recommends immediate investigation for CDR diagnosis by direct microscopy in blood, other fluids or stained tissue [5,13,15,16,21], even in the absence of signs and symptoms. In fact, prospective studies reported early diagnosis of oligosymptomatic cases with better prognosis [15,35,36]. It is noteworthy, however, that factors associated with mortality by CDR and other causes in co-infection have not been shown.

Despite its relevance, data about clinical and epidemiological patterns in patients co-infected with *T*. *cruzi* and HIV are limited to a few single-center studies. In this context, a “Network for Healthcare and Study of *Trypanosoma cruzi*-HIV Co-infection and other immunosuppression conditions” was created in Brazil aiming to provide comprehensive care and study of all the cases [10]. The present international multicenter study was proposed to evaluate the main clinical characteristics and mortality causes of *T*. *cruzi*-HIV co-infected patients, aiming at establishing strategies for a high-quality healthcare and surveillance.

## Methods

### Ethics statement

Data from medical records were anonymously analyzed and the study was approved by Institutional Review Board of research centers (protocol number 095/1995 at the coordinating center, Ethics and Research Committee of Hospital das Clínicas, Faculdade de Medicina, University of São Paulo, Brazil). Formal written consent was obtained from patients except for retrospective study participants who dropped out of follow-up.

### Study design

This is a cross-sectional retrospective multicenter study involving 241 patients with *T*. *cruzi*—HIV co-infection from 13 health centers: 9 from Brazil, 2 from Spain, one from Argentina, and one from Chile, that were notified to the Network between 2007 and 2017 (S1 Table). Clinical-epidemiological data were collected between 1986 and 2017 using a specific case report form (S1 Text).

### Study population and dataset

All included cases were diagnosed with *T*. *cruzi and* HIV infections, most of them were born in CD endemic regions.

CD was diagnosed through serology or parasitological tests. Two of the following high profile anti-*T*. *cruzi* serological tests were initially applied: ELISA with non-purified epimastigotes or recombinant epi/trypomastigote antigens, indirect hemagglutination and indirect immunofluorescence with epimastigotes antigenic preparation. CD diagnosis was confirmed by two positive serological tests. In case of discordant result, a third confirmatory serology was applied: indirect immunofluorescence with epimastigote antigens or immunoblot with trypomastigotes antigens or chemiluminescence with chimeric recombinant antigens. Indirect parasitological enrichment methods (blood culture or xenodiagnoses) were also employed for CD diagnosis and monitoring [2,15,37]. For HIV infection, a positive result by ELISA with HIV1/HIV2 antigens was confirmed by Immunoblot with HIV1/HIV2 antigens). The CDR was diagnosed through direct positive microscopy of parasites in the blood or other biological fluids employing methods for concentration (Blood—microhematocrit, Strout, buffy coat and centrifuged fluid samples) and/or amastigotes forms in stained tissular inflammatory lesions [2,15].

The report form filled by the physicians included sociodemographic, epidemiological and clinical data related to *T*. *cruzi and* HIV infections (S1 Text) by time of each infection diagnosis (CD and HIV), CDR diagnoses, clinical presentation of CD, CD4^+^ T lymphocytes, HIV load, Polymerase Chain Reaction (PCR) for *T*. *cruzi* [22,38], CDR treatment and previous CD treatment. Data were submitted and validated by the researcher from each center. Duplicate cases were excluded from the analysis.

### Statistical analysis

Descriptive statistics comprised median (interquartile range 25%-75%) for continuous variables and frequencies (percentages) for categorical variables. Comparisons between groups according to reactivation status were performed using the Mann-Whitney test for continuous variables and Fisher exact test for categorical variables. Comparisons for CD4^+^ counts measured before and after reactivation were performed using the Wilcoxon matched-pair test. Comparisons between groups presenting CDR according to their status at notification of the case to the *T*. *cruzi*/HIV co-infection Network were performed using Kruskal-Wallis test followed by Dun’s test for multiple comparisons. Additional analyses were performed dividing the sample into two groups according to the time of co-infection diagnosis (before and after 1997, when ART therapy was introduced in Brazil) using the Mann-Whitney test for continuous variables and Fisher exact test for categorical variables. Correlations between continuous variables were obtained by the Pearson coefficient.

The associations between clinical and demographical variables with reactivation in all patients included in the study and between clinical and demographical variables with death among those CDR patients were studied using logistic regression models. Variables that presented a *p*-value<0.20 in the univariate model were included in the multivariate model. The backwards method was used to remove variables that presented a *p*-value>0.05 in the multivariate analysis, until the final model was obtained.

## Results

The S1 Table shows the distribution of the 241 patients according to the centers (170 non-reactivated *T*. *cruzi*/HIV co-infection, 60 with CDR in AIDS and 11 unknown reactivation occurrences). Most (86.7%) were from Brazilian centers.

Table 1 depicts the characteristics of *T*. *cruzi/*HIV patients included in the study. Fifty-five percent of patients were male with a median age of 41.0 years (IQR 25%-75% 34–52) for the entire sample, 40.0 years for males (IQR 25%-75% 33–51), and 45.0 years (IQR 25%-75% 36.5–53) for females (age comparison for male *vs* female, p = 0.05). The CD cardiac and indeterminate forms were the most prevalent, with the median CD4^+^ at co-infection diagnosis of 217 cells/μL and median viral load at co-infection diagnosis of 17,000 viral copies/μL. Almost half of patients (48.1%) were monitored by indirect parasitological enrichment methods. The outcome was death in 34.4% of cases. The interval between co-infection and reactivation diagnoses was ≤ 7 days in 50.0% and simultaneous in 39.7% of cases. There was a moderate Spearman correlation between CD4^+^ count at co-infection and reactivation diagnoses (r = 0.48).

**Table 1:** Characteristics of *T*. *cruzi/*HIV co-infected cases included in the study (n = 241).

Variable	Median (IQR 25%-75%) or % (n)
**Age at co-infection diagnosis (years; n = 238)**	41 (34–52)
**Sex (%; n = 241)**	
Male	55.2 (133)
Female	44.8 (108)
**CD parasitology and molecular biology results (%)**	
Indirect parasitological methods (n = 116)	
Positive	65.5 (76)
Negative	34.5 (40)
PCR (n = 17)	
Positive	64.7 (11)
Negative	35.3 (6)
**CD and HIV diagnoses (n = 232)**	
Simultaneous	16.4 (38)
HIV first	55.2 (128)
CD first	28.5 (66)
**Clinical CD presentation at co-infection diagnosis (%, n = 202)**	
Indeterminate clinical form **(%, n = 202)**	47.0 (95)
Cardiac disease (%, n = 202)*	47.0 (95)
Digestive disease (%, n = 202)*	16.8 (34)
**CD4** ^+^ count at co-infection** cells/μL; n = 219)	217 (76–410)
**CD4** ^+^ **<200 at co-infection (%, n = 219)**	47.5 (104)
**Viral load at co-infection (viral copies/μL; n = 180)**	17,000 (572.5–198,000)
**CDR (%; n = 230)**	26.1 (60)
**CDR parasitological/histopathological results (%)**	
Direct *T*. *cruzi* detection on blood (n = 53)	62.3 (33)
Direct *T*. *cruzi* detection on other fluids (n = 56)	73.2 (41)
Histopathological (n = 38)	18.4 (7)
**Positive CDR parasitological/histopathological results (%, n = 58)**	
One positive test	63.8 (37)
Two positive tests	32.8 (19)
Three positive tests	3.4 (2)
**Clinical presentation at reactivation (%; n = 60)**	
Meningoencephalitis	58.3 (35)
Myocarditis	16.7 (10)
Meningoencephalitis and myocarditis	13.3 (8)
“Others”	11.7 (7)
**Time between co-infection diagnosis and reactivation (months; n = 58)**	0.32 (0–12.8)
**Time between co-infection diagnosis and reactivation (n = 58)**	
Simultaneous	39.7 (23)
≤ 7 days	50.0 (29)
> 7 days	50.0 (29)
**CD4** ^+^ count at reactivation*** (cells/μL; n = 51)	67 (19–202)
**CD4** ^+^ **<200 at reactivation (%, n = 51)**	74.5 (38)
**VL at reactivation (viral copies/μL; n = 33)**	199,000 (50,262–500,000)
**Antiparasitic treatment of reactivation (%; n = 60)**	
Yes	81.7 (49)
No	18.3 (11)
**Status at notification (%; n = 241)**	
Death	34.4 (83)
Active follow-up	43.6 (105)
Lost to follow-up	22.0 (53)
**Time between co-infection diagnosis and death (months; n = 79)**	8.7 (0.9–42.45)
**Time between reactivation and death (months; n = 38)**	1.1 (0.3–2.9)
**Cause of death (n = 83)**	
CDR	28.9 (24)
Chronic CD	9.6 (8)
Other opportunistic AIDS infection	27.7 (23)
Other	18.1 (15)
Not available	15.7 (13)

CD: Chagas disease; CDR: Chagas disease reactivation; HIV: human immunodeficiency virus; IQR: interquartile interval; Indirect parasitological methods = blood culture and/or xenodiagnoses; VL: viral load, PCR: polymerase chain reaction; VL: viral load; “others”: milder oligosymptomatic febrile diseases, myelitis, erythema nodosum and a puerperal patient with *T*. *cruzi* infected newborn;

*Mixed clinical presentation (10.8%; n = 22);

Median CD4^+^ levels similar between men and women at co-infection** (247.5 vs 188.0; *p* = 0.19) and at reactivation*** (75.5 vs 67.0; *p* = 0.31), respectively.

There were no differences for the percentage of reactivation (p = 0.17) or death (p = 0.77) comparing the co-infection diagnosis before (n = 66) or after 1997 (n = 171). However, a statistically significant difference for the time between co-infection and reactivation (before 1997 median time of 11.8 months *vs* after 1997 median time of 0.07 months; p = 0.01) was found. No differences for the time between co-infection and death (p = 0.10) and for the time between reactivation and death (p = 0.72) were observed.

Table 2 depicts the sociodemographic and clinical characteristics according to the presence of CDR. Lower CD4^+^ cells/μL and higher viral load/μL were found among CDR patients in comparison to non-reactivation group at the co-infection diagnosis.

**Table 2:** Characteristics of patients included in the study according to Chagas disease reactivation (n = 230).

Variables	Median (IQR 25%-75%) or % (n)		*p-value* *
	Chagas Disease Reactivation		
	Yes (n = 60)	No (n = 170)	
**Age (years; n = 58 and n = 169)**	41 (34–50)	42 (34–53)	0.79
**Sex (%; n = 60 and n = 170)**			
Male	60.0 (36)	53.5 (91)	0.45
Female	40.0 (24)	46.5 (79)	
**Clinical CD presentation (%, n = 192)**			
Indeterminate form (%, n = 48 and n = 144)	47.9 (23)	49.3 (71)	0.99
Cardiac disease (%, n = 48 and n = 144)**	47.9 (23)	43.8 (63)	0.62
Digestive disease (%, n = 48 and n = 144)**	14.6 (7)	18.1 (26)	0.66
**CD4** ^+^ **count at co-infection (cells/μL; n = 51 and n = 163)**	73 (26–241)	260 (121–443)	<0.001
**CD4** ^+^ **<200 at co-infection (%, n = 51 and n = 163)**	68.6 (35)	40.5 (66)	<0.001
**VL at co-infection (viral copies/μL; n = 37 and n = 138)**	137,756 (21,400–456,000)	7,856 (104–84,500)	<0.001
**Status at notification (%; n = 60 and n = 170)**			
Death	66.7 (40)	20.6 (35)	<0.001
Active follow-up	18.3 (11)	55.3 (94)	
Lost to follow-up	15.0 (9)	24.1 (41)	
**Time between co-infection diagnosis and death (months; n = 36 and n = 35)**	4.3 (0.6–13.0)	19.3 (2.2–62.9)	0.007
**Cause of death (n = 40 and n = 35)**			
**CDR**	60.0 (24)	0 (0.0)	<0.001ƚ
**Chronic CD**	0.0 (0)	20.0 (7)	
**HIV opportunistic infection**	17.5 (7)	42.9 (15)	
**Other**	12.5 (5)	25.7 (9)	
**Ignored**	10.0 (4)	11.1 (4)	
**Time between co-infection diagnosis and death by cause of death (months)**			
**CDR**	0.7 (0.3–8.7)***	---	---
**Chronic CD**	---	5.8 (1.9–76.8)****	---
**HIV opportunistic infection**	9.9 (5.4–15.2)***	5.8 (0.9–47.9)****	0.57
**Other causes**	13.1 (4.8–81.2)***	63.1 (24.3–88.4)****	0.26

CD: Chagas disease; CDR: Chagas disease reactivation; HIV: human immunodeficiency virus; IQR: interquartile interval; VL: viral load.

*Mann Whitney test for continuous and Fisher exact test for categorical variables.

**Mixed clinical presentation (n = 21, 16 non reactivated and 5 reactivated group).

^ɫ^ Reactivation *vs* non-reactivation for CDR (60.0% vs 0.0%; p<0.001) and HIV opportunistic infection (17.5% vs 42.9%; p = 0.02);

Differences among median death times for several causes:

***p = 0.03 -comparison among causes of death only in the reactivation group and

**** *p* = 0.09 –the same, in the non-reactivation group.

At the notification time, active follow-up is more frequent in non-reactivation group than in CDR. Death was more frequent in the reactivation than in the non-reactivation group (Table 2). Similarly, significant differences were shown concerning the main cause of death, the median time between co-infection diagnosis and death and median time between co-infection diagnosis and death by different causes in the reactivation.

Table 3 depicts the characteristics of CDR patients according to the status at notification (n = 60). Among these patients, meningoencephalitis is the most observed presentation (Table 3, n = 35, 58.3%) and seven more benign cases were classified as “others” (two mild oligosymptomatic febrile cases; one oligosymptomatic patient with gastritis, one patient with erythema nodosum, a puerperal patient with *T*. *cruzi* infected newborn, and one febrile case concomitantly to a Hodgkin lymphoma chemotherapy, a patient with myelitis limited to T3-T6).

**Table 3:** Characteristics of patients that presented Chagas disease reactivation according to status at notification (n = 60).

Variable	Median (IQR 25%-75%) or % (n)			*p-value* *
	Status at notification			
	Death (n = 40)	Being Followed (n = 11)	Lost to Follow-Up (n = 9)	
**Age (years; n = 38 and n = 11 and n = 9)**	41 (33–51)	40 (34–45)	46 (41–48)	0.56
**Sex (%; n = 60)**				
Male	70.0 (28)	36.4 (4)	44.4 (4)	0.09
Female	30.0 (12)	63.6 (7)	55.5 (5)	
**Clinical CD presentation (%; n = 48)**				
Chronic Indeterminate form (%; n = 48)	37.9 (11)	70.0 (7)	55.6 (5)	0.20
Cardiac disease** (%; n = 48)	58.6 (17)	30.0 (3)	33.3 (3)	0.20
Digestive disease** (%; n = 48)	13.8 (4)	20.0 (2)	11.1 (1)	0.86
**CD4** ^+^ **count at co-infection (cells/μL; n = 31 and n = 11 and n = 9)**	37 (19–87)	266 (84–464)	228 (56–298)	0.003
**CD4** ^+^ **<200 at co-infection (%, n = 31 and n = 11 and n = 9)**	87.1 (27)	36.4 (4)	44.4 (4)	0.001
**VL at co-infection (viral copies/μL; n = 19 and n = 11 and n = 7)**	188,788 (59,900–294,286)	50,262 (1,100–1,400,000)	195,692 (2,000–1,400,000)	0.54
**Reactivation clinical presentation (%; n = 60)**				
Meningoencephalitis^1^ (n = 35)	67.5 (27)	36.4 (4)	44.5 (4)	0.008
Myocarditis^2^ (n = 10)	12.5 (5)	9.1 (1)	44.5 (4)	
Meningoencephalitis and Myocarditis^3^ (n = 8)	15.0 (6)	9.1 (1)	11.1 (1)	
“Others” ^4^ (n = 7)	5.0 (2)	45.4 (5)	0.0 (0)	
**Time between co-infection diagnosis and reactivation Time (months; n = 38, n = 11 and n = 9)**	0.05 (0–7.9)	45.1 (0–99.2)	0.6 (0.2–4.6)	0.07
**CD4** ^+^ **at reactivation (cells/μL; n = 31, n = 11 and n = 9)**	38 (10–76)	171 (29–340)	240 (55–501)	0.007
**CD4** ^+^ **<200 at reactivation (%, n = 31, n = 11 and n = 9)**	90.3 (28)	54.6 (6)	44.4 (4)	0.004
**VL at reactivation (viral copies/μL; n = 17, n = 9 and n = 7)**	244,037 (59,900–456,000)	68,901 (26,050–355,962)	87,574 (2,000–1,400,000)	0.64
**Antiparasitic treatment (%, n = 60)**				
Yes	85.0 (34)	81.8 (9)	66.7 (6)	0.46
No	15.0 (6)	18.2 (2)	33.3 (3)	
**Time between co-infection diagnosis and death (months; n = 36)**	4.3 (0.6–13.0)	---	---	---
**Time between reactivation and death (months; n = 38)**	1.1 (0.3–2.9)	---	---	

CD: Chagas disease; CDR: Chagas disease reactivation; HIV: human immunodeficiency virus; IQR: interquartile interval; VL: viral load; “Others”: milder oligosymptomatic febrile diseases, myelitis, erythema nodosum and a puerperal patient with *T*. *cruzi* infected newborn;

*Kruskal-Wallis test for differences in continuous variables and Fisher exact test for differences in categorical variables;

** Mixed clinical presentations (n = 5, death = 3, being followed = 2, lost to follow-up = 0). 1vs 4(*p = 0*.*002)*.

Most of the 24 CDR deaths (66.7%) occurred among those with short interval between co-infection and reactivation diagnoses (≤ 5 days), while the remaining occurred with an interval > 2 months. No differences of anti-parasitic treatment were shown between the group that died or was followed. Of note, out of 18 untreated and treated patients who died by CDR with known information between the time from reactivation and death, 5.6% died in the first day after the diagnosis, 11.1% < 7 days, 33.6% <15 days, and 72.2% up to 30 days after the diagnosis of reactivation.

Under antiparasitic treatment, most patients with other milder forms and few severe cases with encephalitis survived. All untreated patients whose death cause was meningoencephalitis died up to the day after the reactivation diagnosis.

Patients who died had lower CD4^+^ cells/μL counts and higher frequency of CD4^+^ count <200/μL at the co-infection diagnosis and at the reactivation time. There were no differences for the comparison of CD4^+^ cells/μL at co-infection and reactivation for the 51 patients that presented the information of CD4^+^ cells/μL at the reactivation time (p = 0.36). In CDR with central nervous system (CNS) involvement, a higher percentage of deaths (Table 3), and lower level of CD4^+^ cells/μL at the reactivation time were registered over myocarditis and “others” presentations (Table 4).

**Table 4:** Distribution of patients according to category of CD4^+^ cells/μL and site of Chagas disease reactivation.

CD4 ^+^ cells/μL	Meningoencephalitis^1^	Myocarditis^2^	“Others”^3^	Meningoencephalitis + Myocarditis^4^	Total
≤100	22	3	2	5	32
101–200	3	1	1	1	6
201–350	2	3	3	0	8
>350	2	2	1	0	5
Number	29	9	7	6	51
Median	29.0	240.0	241.0	62.0	*p = 0.004
25–75% IQR	10.0–73.0	91.0–327.0	43.0–240.0	24.0–100.0	
Min-Max	4–501	30–839	12–537	4–102	

“Others”: milder oligosymptomatic febrile diseases, limited myelitis, erythema nodosum and a puerperal patient with *T*. *cruzi* infected newborn.

*1vs 2 (*p* = 0.0009); 1vs 3 *(p* = 0.008); 2 vs 4 (*p* = 0.02) by Kruskal-Wallis test, followed by Dunn test. IQR—interquartile interval.

Table 5 shows the univariate and multivariate logistic regression analyses to assess the associations between patients’ characteristics and reactivation or death (only among CDR patients). The CD4^+^ count at co-infection was the only variable independently associated with reactivation whereas male sex and CD4 count at co-infection were independently associated with death in CDR patients (Table 5).

**Table 5:** Logistic regression model for association between clinical and demographical variables with reactivation outcome (n = 230) and death among those patients that presented CDR reactivation (n = 51).

**Variables**	**Reactivation (n = 230)**			
	**Univariate**		**Multivariate**	
	**OR (95% CI)**	***p-value***	**OR (95% CI)**	***p-value***
Age (n = 227)	0.99 (0.97–1.02)	0.78	---	---
Male (n = 230)	1.30 (0.72–2.37)	0.39	---	---
CD chronic indeterminate clinical form (n = 192)	0.95 (0.49–1.82)	0.87	---	---
Cardiac clinical form (n = 192)	1.18 (0.61–2.28)	0.62	---	---
Digestive clinical form (n = 192)	0.77 (0.31–1.92)	0.58	---	---
CD4^+^ at co-infection (n = 214)	0.99 (0.99–0.99)	<0.001	0.99 (0.99–0.99)*	0.008
CD4^+^ <200 at co-infection (n = 214)	3.21 (1.65–6.28)	0.001	---	---
VL at co-infection (n = 175)	1.01 (1.00–1.01)	0.01	---	---
**Variables**	**Death (n = 51)**			
	**Univariate**		**Multivariate**	
	**OR (95% CI)**	***p-value***	**OR (95% CI)**	***p-value***
Age (n = 49)	1.02 (0.96–1.08)	0.48	---	---
Male (n = 51)	4.08 (1.01–16.60)	0.04	8.98 (1.11–72.29)	0.04
CD chronic indeterminate clinical form (n = 39)	0.26 (0.06–1.23)	0.09	---	---
Cardiac disease (n = 39)	3.31 (0.71–15.44)	0.13	---	---
Digestive disease (n = 39)	0.64 (0.10–4.17)	0.64	---	---
Reactivation Presentation				
“Others” **	1.0 (Reference)		1.0 (Reference)	
Meningoencephalitis	16.9 (2.4–118.3)	0.004	---	---
Myocarditis	12.5 (0.84–186.3)	0.07	---	
Meningoencephalitis and Myocarditis	15.0 (1.03–218.3)	0.04	---	---
Parasitological treatment	1.35 (0.22–7.33)	0.80	---	---
Co-infection diagnosis to reactivation time (months) (n = 49)	0.97 (0.95–0.99)	0.01	---	---
CD4^+^ at co-infection (n = 42)	0.99 (0.98–0.99)	0.004	0.99 (0.98–0.99)	0.006
CD4^+^ <200 at co-infection (n = 42)	11.81 (2.35–59.46)	0.003	---	---
CD4^+^ at reactivation (n = 42)	0.99 (0.98–0.99)	0.004	---	---
CD4^+^ <200 at reactivation (n = 42)	7.78 (1.45–41.78)	0.02	---	---
VL at co-infection (n = 30)	0.99 (0.99–1.00)	0.31	---	---
VL at reactivation (n = 26)	1.00 (0.99–1.00)	0.43	---	---

*95% CI (0.99465–0.99918);

**“others”: milder oligosymptomatic febrile diseases, myelitis, erythema nodosum and a puerperal patient with *T*. *cruzi* infected newborn

## Discussion

This article addresses an unprecedented case series of 241 patients with *T*. *cruzi* HIV co-infection, 102 previously published as single case report or case series [15,17,19,21,22,35,36,39–41]. The observed rate of CDR in the present study (26.1%) is greater than the previous reported values, that can be explained by the lack of uniformity in the notification of cases, from previous studies and some health centers. Data from reported retrospective studies are variable [5,21], and although CDR is a rare event, the data of patients from large prospective studies could lead to a more reliable rate of CDR [6,15].

Concerning CDR diagnosis, parasites search in the blood by microscope concentration methods is easily performed but the result could be negative in myocarditis or encephalitis, and the search for parasites in other fluids (pericardial and cerebrospinal) or even invasive procedure with biopsy could increase the CDR diagnosis rate [6,15].

In this work, monitoring for CDR was made in 48.1% of patients with co-infection by parasitological enrichment methods, and more rarely, by PCR. Quantitative PCR represents a safety test to monitor parasitemia in immunocompromised hosts when able to differentiate between higher parasitemia in CDR and lower parasitemia in non-reactivation episodes of the co-infection [22,38,42,43]. Therefore, its implementation is essential for CDR detection as rapidly as possible in order to adapt immunosuppressive treatment and to start the anti-parasitic treatment.

Our data demonstrated new features on morbidity and mortality of *T*. *cruzi*/HIV co-infection besides interferences of CDR in the natural history of *T*. *cruzi/*HIV co-infection. Among these evidences generated by our study are: a shorter time between co-infection diagnosis and death in reactivation than in non-reactivation patients by multivariate analysis (p = 0.006); death status at notification registered more frequently in the reactivation group while “being followed” status in the non-reactivation group (p<0.001); different causes of death between reactivation (predominance of CDR) and non-reactivation group (mainly opportunistic AIDS infections) (p<0.001) with different evolution and outcomes; higher percentage of deaths in meningoencephalitis and lower in “others” non-severe cases of reactivation (p = 0.008); and CD4 counts differences among several sites of reactivation (p<0.004), with lower CD4^+^ counts in meningoencephalitis or “meningoencepahlitis + myocarditis” compared to myocarditis, as well as in meningoencephalitis compared to “others” (p = 0.0009, p = 0.008, and p = 0.02, respectively).

We emphasized that the statistical difference of time lapsed from co-infection diagnosis to CDR or death showed a clear gap about opportunity of timely diagnose both *T*. *cruzi* and HIV infections in patients under follow-up for early detection of CDR. In addition, this association emphasizes the need of careful management of both infections by controlling adherence to ART and monitoring *T*. *cruzi* parasitemia soon after co-infection diagnosis.

In our study, HIV infection was the first diagnosed infection in 55.2% of all co-infection cases, and simultaneous diagnosis of HIV and CD only occurred in 16.4% of cases. Remarkably, about 50% of co-infection cases were diagnosed only 7 days before reactivation diagnosis, emphasizing the need for active search for CD when HIV was first diagnosed and vice-versa upon clinical and epidemiological suspicion and serological testing or even in the absence of signs and symptoms. In fact, the greater interval between co-infection and reactivation diagnosis before 1997 is parallel to the initial effort from the “Network for Healthcare and Study of *Trypanosoma cruzi*/HIV co-infection and other immunosuppression conditions” for an early diagnosis of both infections. Significant difference for the time between co-infection to reactivation (higher before 1997, due to active search from physician aware of CD in some centers) with no difference between co-infection diagnosis to death (p = 0.10) and reactivation time to death (p = 0.72) suggest that co-infection diagnosis was early detected in the first period but not enough to modify death, probably due to the lack of timely CDR diagnosis and antiparasitic treatment. After 1997, creation of Brazilian reference centers for HIV/AIDS increased the % of first HIV diagnosis in the co-infection diagnosis. However, CD continues to be a neglected disease and has not been the main focus for HIV specialists. Consequently, co-infection has become a late diagnosis in these centers, close to reactivation diagnosis.

Although it is expected that ART introduction in 1997 decreased the percentage of CDR, these data are only observed in a few centers that described CDR cases before 1997. In other centers inside and outside Brazil, most of the cases occurred after 1997. Unfortunately, about 60% of CDR cases in Brazilian centers and 90% outside Brazil after 1997 were not on ART, both by absence of HIV diagnosis or abandonment of therapy.

Therefore, active search of CD has not been a priority for physicians. Thus, the spread of the need for early diagnosis of co-infection remains a challenge to restore the immune response by ART and control *T*. *cruzi* parasitemia.

In agreement with most published papers, our data recognize the meningoencephalitis as the most common reactivation site, followed by myocarditis, myocarditis and meningoencephalitis and “others” sites [5,15,35,36]. In accordance to its severity and high lethality, we also found that meningoencephalitis was associated with lower levels of CD4^+^ (median = 29 cells/μL) in comparison to those patients classified as “others” forms (median = 241 cells/μL), concomitantly to more benign clinical forms and lower lethality.

The description and evolution of the group classified as “others” brought new information and additional cases to those previously described [15,35,36] as oligosymptomatic and mild disease with higher CD4 counts/μL (Table 4) (Table 3). In fact, we added to previously published oligosymptomatic cases [15,35,36], three more cases presenting with febrile disease concomitantly to a Hodgkin lymphoma, gastritis and T3-T6 myelitis. Other four reported cases had oligosymptomatic febrile disease, erythema nodosum and one is a puerperal oligosymptomatic patient with *T*. *cruzi* infected newborn [15,35,36]. We confirmed here a better outcome in this group, with 71.4% (5 out 7 patients) classified as “others” surviving after antiparasitic treatment in contrast to 12.9% (4 out 31 patients) with meningoencephalitis (Table 3).

In these cases, the diagnosis needs to be actively searched since a better response to antiparasitic treatment has been shown in severe cases [5,15,21,23].

Multivariate logistic regression analysis showed an increased risk of death in male patients with reactivation. This original data can be partially explained by the greater risk of death in males with Chagas heart disease, as documented before in non-HIV infected patients [44]. In patients with CDR who died, cardiac disease occurred in 58.6%. In addition, a Brazilian study of naïve patients for active antiretroviral therapy (ART) reported higher frequency of CD4^+^ counts <200 cells/μL in men than women [45] but our work did not show differences between men and women for median CD4^+^ levels at co-infection and reactivation diagnoses. Moreover, data analyses from mortality in South African patients with CD4^+^ basal and follow-up levels under control showed a 20% higher risk of death for males than females at 24 and 36 months after starting ART [46]. Better immune response to treatment was shown in women with higher increases of CD4^+^ counts, representing a challenge for future studies.

We emphasize the role of higher CD4^+^ counts at the time of co-infection diagnosis as a protective factor for both reactivation and mortality, confirmed by multivariate logistic regression analyses. Although high mortality and CD4^+^ counts lower than 200 or 300 cells/μL have been described in case report or case series of CDR, there was only one previous comparative analysis in the literature [21], that showed a significant association between lower CD4+cells/ μL at co-infection diagnosis and CDR in the univariate analysis [21]. However, these authors recognized the small number of the patients to perform a multivariate analysis. In this setting, our results are partially explained by the short interval between co-infection and reactivation diagnoses in 50% of cases. In our sample, detectable viral loads (median = 199,000 viral copies/μL) and CD4^+^ counts (median = 67 cells/μL) at reactivation diagnosis suggest the lack of effective antiretroviral therapy. In addition, an association of lower CD4^+^ counts at the time of co-infection diagnosis and mortality in the reactivation group was confirmed for the first time to our knowledge using a multivariate analysis. Previous report did not find significant statistical difference concerning this variable [21]. We also observed that about 70% of deaths were reported within 5 days between co-infection diagnosis and death and that a shorter period of time was observed between co-infection diagnosis and death in the reactivation than in the non-reactivation group (p = 0.007). Remarkably, the presence of detectable viral loads (median = 17000 copies/μL) and CD4^+^ cells/μL (median = 217) at co-infection diagnosis in our entire sample of *T*. *cruzi*/HIV infected patients emphasizes the need of early diagnosis of co-infection and introduction of effective antiretroviral therapy.

In fact, CD4^+^ cells play a central role in HIV/AIDS evolution since CD4^+^ count depletion has been linked to cellular immunity impairment and susceptibility to opportunistic infections [47]. Severe lymphopenia, unresponsiveness to *in vivo* and *in vitro* tests, and marked decrease of CD4^+^ cells were reported in HIV infected patients in association with opportunistic infection [47]. This immunodeficiency is attributed to the impairment of CD4^+^-mediated viral killing [48], and is reversed by antiretroviral protease inhibitor [49].

On the other hand, stimulation of CD4^+^ by *T*. *cruzi* antigens induces macrophage activation, interferon γ secretion and increases its ability to kill the parasites [50,51]. In addition, the role of CD4^+^ cells (Th1 response) in protecting against increased *T*. *cruzi* parasitemia has been demonstrated by higher parasitemia in CD4^+^ depleted mice [52,53].

T cells have also been considered as unresponsive to secrete IFNγ and IL2 cytokines under *T*. *cruzi* antigens stimulation in a murine model of infection with parasite and leukemia virus, associated with CDR and higher parasitemia [54]. In parallel, a Th2 response was also reported in association with parasitemia in *T*. *cruzi*/HIV infected patients [31].

Although antiparasitic treatment did not cause mortality differences between treated and non-treated CDR patients, this lack of efficacy can be related to the lack of opportunity to timely receive it. Thus, patients diagnosed too late and presenting severe central nervous system or myocardium involvement do not have opportunity to have the protective effect of antiparasitic treatment.

The present study showed, for the first time, a more comprehensive data about outcome in 49 treated patients with CDR. In fact, a proportion of 72.2% of the total deaths due to CDR occurred by the 30^th^ day after reactivation diagnosis, without the possibility to complete the recommended 60 days of antiparasitic treatment. Among 11 survivor cases, six had meningoencephalitis and five a milder form of reactivation (“others”). In contrast, 11 patients did not receive antiparasitic treatment, the majority with meningoencephalitis. Due to the retrospective nature, the reasons for this absence of treatment were not known and can be attributed to losses of follow-up, lack of drugs accessibility or impossibility of oral drug intake. In all patients who died of meningoencephalitis without antiparasitic treatment, death occurred within 36 hours after the reactivation diagnosis, so early antiparasitic treatment is mandatory in all cases.

This work has limitations mainly due to the variability of the data retrospectively included by the health centers over a 31 years period, at times that differ in terms of access to full treatment for HIV infection (ART not available for the first cases). Differential processes for co-infection cases registration and follow-up routines, low accessibility to indirect parasitological and/or molecular methods may contribute to some issues observed in this study. In addition, although it is the largest known sample, considering the limited size of CDR group and interval between co-infection and reactivation diagnoses, associations of CD4^+^ with reactivation and death need to be confirmed in future studies. Despite these limitations, this study highlights, in 241 *T*. *cruzi*/HIV co-infected patients with 60 reactivations, significant interference of CDR in the natural history of CD and HIV infections. In summary, these novel associations of CD4^+^ cells/μL with both reactivation and death as well as with the meningoencephalitis in CDR emphasize the role of these cells as risk factors for CDR and reinforce the need of a preserved immune response provided by ART in co-infection. Additionally, despite the high lethality, we reinforce the role of early antiparasitic treatment in meningoencephalitis survivors since non-treated severe cases died soon after diagnosis.

This multicenter study emphasizes the crucial need for early diagnosis and immediate treatment of both *T*. *cruzi* and HIV infections, for monitoring HIV evolution under ART, controlling *T*. *cruzi* parasitemia, and applying more efficient and effective tools and protocols. In addition, data gathering for a comprehensive evidence-based patient care, health professionals training and case surveillance are critical to break the underdiagnosis and epidemiological silence.

## Supporting information

S1 Table Distribution of 241 patients with *T*. *cruzi*/HIV co-infection (CO) according to their status of Chagas disease reactivation and to the reference centers. (PDF)

S1 Text Case report forms for *T*. *cruzi*/HIV co-infection patients. A. Portuguese B. English.
(PDF)
