# Correction: Clinical profile and mortality in patients with *T*. *cruzi*/HIV co-infection from the multicenter data base of the “Network for healthcare and study of *Trypanosoma cruzi*/HIV co-infection and other immunosuppression conditions”

**DOI:** 10.1371/journal.pntd.0011036

**Published:** 2023-01-04

**Authors:** Maria Aparecida Shikanai-Yasuda, Mauro Felippe Felix Mediano, Christina Terra Gallafrio Novaes, Andréa Silvestre de Sousa, Ana Marli Christovam Sartori, Rodrigo Carvalho Santana, Dalmo Correia, Cleudson Nery de Castro, Marilia Maria dos Santos Severo, Alejandro Marcel Hasslocher-Moreno, Marisa Liliana Fernandez, Fernando Salvador, Maria Jesús Pinazo, Valdes Roberto Bolella, Pedro Carvalho Furtado, Marcelo Corti, Ana Yecê Neves Pinto, Alberto Fica, Israel Molina, Joaquim Gascon, Pedro Albajar Viñas, Juan Cortez-Escalante, Alberto Novaes Ramos Jr, Eros Antonio de Almeida

There is an error in the last sentence of the seventh paragraph of the Results section. It should read: Of note, out of 18 treated patients who died by CDR with known information between the time from reactivation and death, 5.6% died in the first day after the diagnosis, 11.1% < 7 days, 33.6% <15 days, and 72.2% up to 30 days after the diagnosis of reactivation.
