# Supplementary material for: Clinical profile and mortality in patients with T. cruzi/HIV co-infection from the multicenter data base of the “Network for healthcare and study of Trypanosoma cruzi/HIV co-infection and other immunosuppression conditions”
Source: PLoS Negl Trop Dis. 2021 Sep 30;15(9):e0009809. doi: 10.1371/journal.pntd.0009809 (PMC8483313; doi:10.1371/journal.pntd.0009809)
Supplement: S1 Table — (PDF) [file pntd.0009809.s001.pdf]

S1_Table. Distribution of 241 patients with *T. cruzi*/HIV co-infection (CO) according to their status of Chagas Disease Reactivation and to the reference centers.

Health center	Number of patients				
	No-reactivation (A)	CDR (B)	CDR unknown	Total	% CDR B / (A+B)
A – Faculdade de Medicina, University of São Paulo, São Paulo, Brazil	46	12	0	58	20.7%
B – Faculdade de Medicina Ribeirão Preto, University of São Paulo, São Paulo, Brazil	29	5	4	38	14.3%
C – Federal University of Triangulo Mineiro, Minas Gerais, Brazil	21	16	0	37	43.2%
D – Faculty of Medical Sciences, University of Campinas, São Paulo, Brazil	21	4	6	31	16.0%
E – School of Medicine, University of Brasilia, Brasília, Distrito Federal, Brazil.	17	1	0	18	5.6%
F– Federal University of Health Sciences of Porto Alegre, Porto Alegre, RGS, Brazil	14	0	0	14	0.0%
G–National Institute of Infectious Diseases, Oswaldo Cruz Foundation, Health, Rio Janeiro, Brazil	7	1	1	9	12.5%
H–Vall d'Hebron University Hospital,PROSICS,Universitat Autònoma de Barcelona,Barcelona,Spain	7	0	0	7	0.0%
I – Health Centre n. 8. Brasilia, Distrito Federal, Brazil	3	0	0	3	0.0%
J – Universitat de Barcelona, Hospital Clínic, ISGlobal. Barcelona, Spain.	3	0	0	3	0.0%
K– Hospital de Enfermedades Infecciosas Francisco Javier Muñiz. Buenos Aires, Argentina	1	20	0	21	95.2%
L– Evandro Chagas Institute, Health Surveillance Secretary, Health Ministry, Belém, Pará, Brazil	1	0	0	1	0.0%
M– Infectious Disease Service, Hospital Militar de Santiago, Santiago, Chile.	0	1	0	1	100.0%
**Total**	**170**	**60**	**11**	**241**	26.1%
