# Supplementary material for: Clinical profile and mortality in patients with T. cruzi/HIV co-infection from the multicenter data base of the “Network for healthcare and study of Trypanosoma cruzi/HIV co-infection and other immunosuppression conditions”
Source: PLoS Negl Trop Dis. 2021 Sep 30;15(9):e0009809. doi: 10.1371/journal.pntd.0009809 (PMC8483313; doi:10.1371/journal.pntd.0009809)
Supplement: S1 Text — A. Portuguese B. English. (PDF) [file pntd.0009809.s002.pdf]

S1_Text. Case report forms for *T. cruzi*/HIV co-infection patients

A. **Portuguese**

1 Unidade de Saúde: ____

2 Médico Responsável: ______________________________ **3** Data de Notificação: ___/___/___

4 Diagnóstico Infecção HIV: ___/___/___ **5** Diagnóstico D. Chagas: ___/___/___ **6** Diagnóstico Reativação: ___/___/___

7 Situação no momento da notificação: | | 1=Óbito; 2=Em seguimento; 3=Abandono; 9=Ign **8** Se óbito, data: ____/____/____

9. Causa básica do óbito, conforme interpretação médica |__| 1.reativação d. Chagas |__|2. D.chagas crônica, |__ |3. D. oportunista da aids. |__| 4 +outros 9=Ign

IDENTIFICAÇÃO (dados no momento do diagnóstico da definição da co-infecção)

10 Nome do Paciente: ____

11 Data de Nascimento: : ___/___/___**12** Idade: |48| |__| dias |__| meses |___| anos **13** Sexo: | | **1**=Masc **2**=Fem; **9**=Ignorado

14 Nome da Mãe: ____

15 Local de Nascimento: (município/estado; rural/urbano): ____-____

16 Município de Residência: ____

18 UF: ___________

19 País (se residente fora do Brasil: ____

20 Raça/Cor | | **1**=Branca; **2**=Preta ; **3**=Amarela; **4**=Parda; **5**=Indígena; **6**=Outro; **9**=Ignorado

DADOS CLÍNICOS E LABORATORIAIS INICIAIS (considerar os primeiros 6 meses após diagnóstico de co-infecção)

21 Megaesôfago: |__| **1**=sim; **2**=não; **9**=ign **22** Megacólon: |__| **1**=sim; **2**=não; **9**=ign

23 Cardiopatia: |__| **1**=sim; **2**=não **9**=ign **24** Forma indeterminada: |__| **1**=sim; **2**=não.

25 Provas sorológicas para D. Chagas*: |__| **1**=pos; **2**=neg; **3**=Inconclusivo; **4**=Não foi feito; **5**=Ignorado.

26 Data: ____/____/___

27 Diagnóstico parasitológico: |__| **1**= pesquisa direta (+) *T. cruzi* sangue**; 2** = histopatológico; **3** = pesquisa direta de *T. cruzi* em fluidos associada à histopatologia; **4 =** outros (citar)____ ____; **9** = ignorado.

28 Xenodiagnóstico |__| **1**=pos; **2**=neg; **3**=não realizado; **9**=Igorado

29 Data do primeiro resultado positivo _____/____/___

30 Hemocultura |__| **1**=pos; **2**=neg; **3**=não realizado; **9**=Ign **31** Data do primeiro resultado positivo _____/____/___

32 PCR |__| **1**=pos; **2**=neg; **3**=não realizado; **9**=Ign **33** Data do primeiro resultado positivo ____/____/___

34 Outros |________________________________| **35** Data do primeiro teste ____/____/___

36 Contagem linfocitária: CD4 = ___cels/μL **37** Data da Coleta ____/____/___

38 Carga Viral do HIV _____________________ **39** Data da Coleta ___/____/__

40 O paciente co-infectado: Reativou?: |__| 1=Sim; 2=Não; 9=Ignorado. **(Se houve reativação, pular para 42)**

41 Se não houve reativação, recebeu tratamento específico? |__| 1=Sim; 2=Não; 3=Outros; 9=ignorado

.**(Se não houve reativação, finalizar na 41)**

DADOS CLÍNICOS E LABORATORIAIS NA REATIVAÇÃO
Preencher a seguinte informação nos pacientes que REATIVARAM

42 Tipo de Reativação: |__| **1**= Meningoencefalite; **2**= Miocardite; **3**=Outro (citar); ________________________________ **9**=ignorado.

43 Diagnóstico da Reativação: |__| **1**= pesquisa direta (+) *T. cruzi* sangue**;** **2** = histopatológico; **3** = pesquisa direta de *T. cruzi* em fluidos associada à histopatologia; **4 =** outros (citar) ____**;** **9** = ignorado.

44 Contagem linfocitária: CD4 = ________ cels/μL **45** Data da Coleta ____/____/____

46 Carga Viral do HIV ________ cópias/μL **47** Data da Coleta ____/____/____

48 Uso de benzonidazol? I__I 1-sim; 2-não

Dose de benzonidazol:___________________________________Data: ____/____/____

49 Uso de imidazólicos? I__I 1-sim; 2-não. Especificar qual :________________________

Dose de imidazólico:_____________________________________Data: ____/____/____

INSTRUCIONAL

Definição de caso de co-infecção: Pacientes que apresentam confirmação de infecção concomitante do Vírus da Imunodeficiência Humana e do protozoário *Trypanosoma cruzi.*

Definição de caso de reativação de doença de Chagas: Em paciente imunodeprimido, presença de tripomastigotas de *T. cruzi* diretamente observados ao exame microscópico do sangue periférico, liquor, líquido pericárdico, ou de outros fluidos corporais, ou ocorrência de alterações histopatológicas compatíveis com processo inflamatório agudo e presença de ninhos de amastigotas.

Provas sorológicas para Doença de Chagas: Considerar ELISA e/ou R. Imunofluorescência indireta e/ou Hemaglutinação e/ou Quimioluminescência ou prova sorológica de reconhecida sensibilidade/especificidade.

*Instrucional: + = 2ou 3 positivos em 2 ou 3. Negativo 2 de 2 ou 3 de 3 negativos. Inconclusivo: nem + nem Negativo. NFF não foi feito

Prova sorológica para HIV – Elisa positiva, seguida de confirmação por Imunoblot.

Estágios do comprometimento cardíaco na Cardiopatia Chagásica Crônica

Estágios	Eletrocardiograma	Ecocardiograma	Insuficiência cardíaca congestiva
A	Alterado	Normal	Ausentet
B1	Alterado	Alterado,. FEVE ≥45%	Ausente
B2	Alterado	Alterado, FEVE < 45%	Ausente
C	Alterado	Alterado	Compensada
D	Alterado	Alterado	Refractária

Classificação do comprometimento esofàgico (Rezende, 1980)

Grupos	Alterações no esôfago
1	Calibre normal ao RX. Trânsito lento de contraste
2.	Calibre com aumento leve a moderado. Considerável retenção de contraste. Ondas terciárias frequentes associadas ou não à hipertonia esofágica.
3	Grande aumento de diâmetro. Atividade muscula diminuída. Hipertonia do esôfago inferior. Grande retenção do contraste..
4.	Dolicomegaesôfago. Capacidade de retenção aumentada, atônico, alongado, curvando-se sobre a cúpula diafragmática.

Comentários:

B. English

1-Health Unit____
2-Doctor Responsible____
3-Notification Date _____/____/_____
4-HIV Infection diagnosis ____/____/____
5-Chagas disease diagnosis _____/____/____
6- Reactivation diagnosis ____/____/____
7-Clinical outcome at the time of notification: (   ) 1-death; 2-follow-up; 3-loss of follow-up; 9-ignored
8- In case of death, Date ____/____/____
9- Basic cause of the death, according to medical interpretation: (   ) 1-reactivation of Chagas disease; 2-chronic Chagas disease; 3-AIDS-associated opportunistic infections; 4-others; 9-ignored

IDENTIFICATION (at the moment of coinfection diagnosis)

10-Patient's Name____
11-Date of birth   ___/___/___
12-Age ___/____ days _____ months ____
13-Sex (   ) female   (   ) male
14-Mother´s Name ____
15-Birth place (municipality/state; rural/urban) _______________________________
16-Municipality of residence_______________________________________
17-State____
18- State abbreviations ____
19-Country (if you live outside of Brazil)) __________________________________
20- Race / color (   ) 1-white; 2-black; 3-yellow; 4-indigenous; 5-other; 9-ignored

INITIAL CLINICAL AND LABORATORY DATA

(consider the first six months after the diagnosis of coinfection)

21- Megaesophagus (   ) 1-Yes; 2-No; 9-ignored
22- Megacolon (   ) 1-Yes; 2-No; 9-ignored
23-Cardiopathy (   ) 1-Yes; 2-No; 9-ignored
24-Indeterminate form (   ) 1-Yes; 2-No
25-Serological tests for Chagas' disease*: (   ) 1-Positive; 2- Negative; 3-Inconclusive; 4- Not done; 5-Ignored______________________26- Date____/____/____
27-Parasitological Diagnosis: (   ) 1-Direct search and *Trypanosoma cruzi* observed in blood; 2-Histopathological; 3-Direct search of *Trypanosoma cruzi* observed in fluids associated with histopathology; 4- Other findings (complete)___________________9. Ignored 26- Date____/____/____
28-Xenodiagnosis: (   ) 1-positive; 2- Negative; 3-Unrealized; 9- Ignored . 29- Date of first positive result ___/____/____

30- Blood culture: ( ) 1-positive; 2- Negative; 3-Unrealized; 9- Ignored.<br>31- Date of first positive result (Blood culture) ___/____/____
32-PCR ( ) 1-positive; 2- Negative; 3-Unrealized; 9- Ignored.<br>33- Date of first positive result (PCR) ___/____/____
34- Others_____________________________________35- Date of first test ___/____/____
36- CD4 count (coinfection) = _______________cells/ µL 37- Collection date ___/____/____
38- HIV viral load (coinfection) = ____________ copies/ µL 39- Collection date___/____/____
40- Was the coinfected patient reactivated? ( ) 1-Yes; 2-No; 9-Ignored<br>**(If reactivation occurs, skip to 42)**
41- If there was NOT reactivation, did patient receive specific treatment? ( ) 1-Yes; 2-No; 9-ignored<br>(**If there was no reactivation, finish at 41)**

CLINICAL AND LABORATORY DATA ON REACTIVATION

Complete the following information from patients with reactivation - consider the first 6 months after the diagnosis of reactivation

42-Reactivation type: ( ) 1-Meningoencephalitis; 2-Myocarditis; 3-Other findings (complete)____; 9-ignored
43- Reactivation Diagnosis: ( ) 1-Direct search and *Trypanosoma cruzi* observed in blood; 2-Histopathological; 3-Direct search of *Trypanosoma cruzi* observed in fluids associated with histopathology; 4- Other findings (complet)______________9. Ignored
44- CD4 count (reactivation) = __________cells/µL 45- Collection date___/____/____
46- HIV viral load (reactivation) = _______ copies/µL 47- Collection date___/____/___
48- Treatment: ( ) 0 – no treatment 1- benznidazole; 2- nifurtimox<br>Dose _______ Duration:_________ (days) Date of beginning ____/____/____
49- Use of imidazole? ( ) 1-Yes; 2-No. Imidazole name: ________________________<br>Dose of imidazole _______ Date: ____/____/____

INSTRUCTIONAL

Co-infection case definition: Patients with confimation of concomitant infections by *Trypanosoma cruzi.a*nd HIV.

Case definition of Chagas disease Reactivation: Presence of T. cruzi trypomastigotes under direct microscopy on peripheral blood, cerebrospinal fluid, pericardial fluid or other body fluids,(or occurrence of histopathological changes compatible with acute inflammatory proccess with amastigote nests.

Serology: ELISA, Indirect Immunofluorescence, Chemoluminescence, Hemagglutination and/or high performance serological test. (sensitivity/specificity).

*Instructional: + = 2 or 3 positives in a total of 2 or 3. Negative: 2 of 2 or 3 of 3 negatives. Inconclusive: neither positive or negative. NFF non performed

---

Stages of myocardium involvement in Chronic Chagasic cardiopathy

---

Stages	Electrocardiogram	Ecocardiogram	Heart Failure
A	With changes	Normal	Absent
B1	With changes	Changes. FEVE ≥45%	Absent
B2	With changes	Changes FEVE < 45%	Absent
C	With changes	Changes	Compensable
D	With changes	Changes	Refractory

Classification of esophageal involvement (Rezende, 1980)

Groups	Changes in the esophagus
I	Normal diameter on RX. Slow contrast transit
2.	MIld to moderate increase of diameter. Considerable contrast retention. Frequent tertiary waves associated or not with esophagus hipertony.
3	Great diameter increase. Decresead muscular activity. Inferior esophagus hypertonic. Big contrast retention.
4.	Dolichomegaesophagus. Increase retention capacity, atonic and elongated., curving over the diaphragmatic dome

Comments:
